# Ordered arrays of nanoporous silicon nanopillars and silicon nanopillars with nanoporous shells

**DOI:** 10.1186/1556-276X-8-42

**Published:** 2013-01-21

**Authors:** Dong Wang, Ran Ji, Song Du, Arne Albrecht, Peter Schaaf

**Affiliations:** 1 Materials for Electronics, Institute of Materials Engineering and Institute of Micro- and Nanotechnologies MacroNano®, Ilmenau University of Technology, Gustav-Kirchhoff-Str. 5, Ilmenau, 98693, Germany; 2 SÜSS MicroTec Lithography GmbH, Schleissheimer Str. 90, Garching, 85748, Germany; 3 Center for Micro- and Nanotechnologies MacroNano®, Ilmenau University of Technology, Gustav-Kirchhoff-Str. 7, Ilmenau, 98693, Germany

**Keywords:** Nanoporous Si, Pillars, Nanowires, Metal-assisted chemical etching, Nanoimprint lithography

## Abstract

The fabrication of ordered arrays of nanoporous Si nanopillars with and without nanoporous base and ordered arrays of Si nanopillars with nanoporous shells are presented. The fabrication route is using a combination of substrate conformal imprint lithography and metal-assisted chemical etching. The metal-assisted chemical etching is performed in solutions with different [HF]/[H_2_O_2_ + HF] ratios. Both pore formation and polishing (marked by the vertical etching of the nanopillars) are observed in highly doped and lightly doped Si during metal-assisted chemical etching. Pore formation is more active in the highly doped Si, while the transition from polishing to pore formation is more obvious in the lightly doped Si. The etching rate is clearly higher in the highly doped Si. Oxidation occurs on the sidewalls of the pillars by etching in solutions with small [HF]/[H_2_O_2_ + HF] ratios, leading to thinning, bending, and bonding of pillars.

## Background

Nanostructured Si is drawing a great deal of interest due to its potential applications in nanoscale electronics [1,2], optoelectronics [3], thermoelectrics [4], photovoltaics [5], biosensors [6], nanocapacitor arrays [7], and as electrodes in Li-ion batteries [8]. It is well known that porous Si can be produced by anodic (electrochemical) etching in HF aqueous solution or stain etching in HNO_3_/HF solution [9,10]. Recently, metal-assisted chemical etching (MaCE) as a simple and low-cost method to fabricate Si nanowires and nanoporous Si has attracted increasing attention [11-14]. In this process, Si wafer coated with a noble metal is etched in a solution consisting of HF and an oxidant (e.g., H_2_O_2_ or AgNO_3_) to form the nanostructures. Nanoparticles or thin films of noble metals (e.g., Au, Ag, or Pt) are used to catalyze the etching. Two-level nanoscaled porous Si nanowires were even synthesized with highly doped Si using MaCE, and Ag nanoparticles acted as catalyst [15-17]. Zigzag Si nanowires were fabricated with (111)-oriented Si by MaCE (with Ag nanoparticles as catalyst) [18]. These zigzag Si nanowires were even fabricated with (100)-oriented Si by a two-step MaCE (with Au film as catalyst) [19]. In general, the structural properties and morphologies of the nanostructured Si produced by MaCE are affected by three main factors: (1) the properties of the deposited noble metals, including the type and form of the metal, and its deposition method; (2) the properties of the Si wafer, including the doping type and level and the crystallographic orientation; and (3) the properties of the etchant, including etchant composition, concentration, and temperature.

By combining MaCE with nanolithography, many ordered nanostructures were fabricated. For example, ordered arrays of Si nanowires and nanopillars were fabricated using a combination of laser interference lithography or nanosphere lithography and MaCE [20-22]. An Au/Ag bi-layer metal mesh with an array of holes, prepared from an anodic aluminum oxide membrane, was used to fabricate Si nanowires by MaCE [23]. In this paper, the fabrication of ordered arrays of nanoporous Si nanopillars, ordered arrays of nanoporous Si nanopillars with nanoporous base, and Si nanopillars with nanoporous shells using a combination of substrate conformal imprint lithography (SCIL) and MaCE (with Au film as catalyst) is presented. The mechanisms of MaCE are systematically investigated, and the fabricated nanoporous pillars should have the potential for applications in sensors and optoelectronics.

## Methods

**Figure 1:**
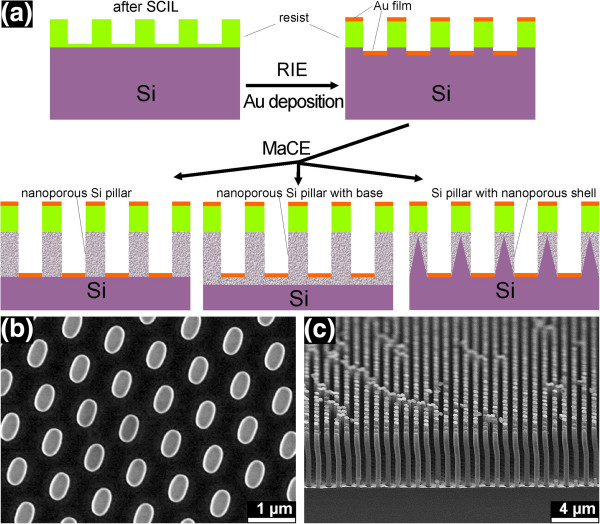
**Fabrication of the ordered arrays of Si nanopillars. (a)** Schematic sketch of the fabrication process for the ordered array of nanoporous Si nanopillars, ordered array of nanoporous Si nanopillars with nanoporous base layer, and ordered array of Si nanopillars with nanporous shells. **(b)** SEM image of the pattern defined using SCIL. **(c)** SEM image of the formed Si nanopillars for the lightly doped Si wafer after MaCE (in *λ*_3_ solution for 10 min).

The fabrication process is schematically represented in Figure 1a. As shown in Figure 1b, an array of elliptical pillars with hexagonal symmetry was defined using SCIL on two types of (100)-oriented p-Si wafers: one is highly doped (B-doped, *ρ* < 0.005 Ω cm), and the other is lightly doped (B-doped, *ρ* = (6.0−10.5) Ω cm). The periodicity (the distance between two adjacent pillars) is 1.0 μm, and the major and minor diameters of the ellipses are 613 and 385 nm, respectively. SCIL was developed by Philips Research and SÜSS MicroTec as a new technique of nanoimprint lithography, and this new technique possesses the advantages of both UV nanoimprint lithography techniques with a rigid stamp for best resolution and with a soft stamp for large-area patterning [24]. Two steps of reactive ion etching (RIE) were performed to transfer the structure into the Si substrate: the residual layer of the resist was removed using inductively coupled plasma RIE, and then the structure was transferred into the Si using RIE. The undercut was etched in the Si after the second-step RIE in order to ensure the separation of subsequently deposited Au films between the pillar areas and the ground areas (as seen in Additional file 1: Figure S1b). A 20-nm-thick Au film was then deposited on the samples using e-beam evaporation. Subsequently, the samples were submerged in a HF/H_2_O_2_ aqueous solution for MaCE. As an example, Figure 1c shows the formed Si nanopillars. The molar proportion of HF/H_2_O_2_/H_2_O is *X*:*Y*:*Z*, where (X + Y):Z is kept constant at 1:5, and the molar ratio *λ* is defined as *λ* = X / (X + Y). The solutions with different molar ratios, *λ*, used in this work are listed in Table 1. During MaCE, only Au contact areas were etched, resulting in vertically aligned arrays of nanoporous Si nanopillars, arrays of nanoporous Si pillars with a nanoporous base, or Si nanopillars with nanoporous shells. The different structural properties are determined by the molar ratio *λ* and the doping level of the Si wafer. After MaCE, the samples were investigated using an ultrahigh resolution scanning electron microscope (SEM; Hitachi S-4800). The resist and the Au film were not removed for SEM inspection.

**Table 1:** **List of solutions with different molar ratios, *λ*, used for the etching solutions**

**Molar ratio**	**Value**
*λ*_1_	0.5
*λ*_2_	0.7
*λ*_3_	0.85
*λ*_4_	0.92

## Results

**Figure 2:**
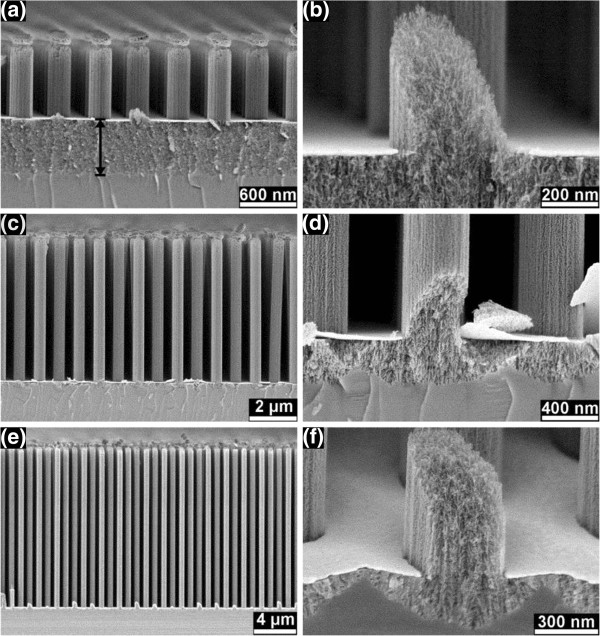
**SEM images of nanopillars formed from the highly doped Si in *λ***_**3 **_**solution.** After etching for 3 min **(a, b)**, 6 min **(c, d)**, and 10 min **(e, f)**, respectively. Panels **b**, **d**, and f show the cracked nanopillars. These cracks were formed during breaking of the samples for the SEM investigation. The distance mark in (**a**) indicates the range of the nanoporous base layer underneath the Au film and the nanopillars.

The nanopillars formed from highly doped Si after etching in *λ*_3_ solution for 3, 6, and 10 min, respectively, are shown in Figure 2. After 3-min etching, the nanoporous Si nanopillars had a vertical length of 1.6 μm, accompanied by the formation of a nanoporous base with a homogenous thickness of 1.2 μm below the Au film and the nanopillars (Figure 2a). After 6-min etching, the length of the nanoporous Si nanopillars increased to 6.3 μm, while the thickness of the nanoporous base is clearly reduced to a few hundred nanometers and not being homogenous anymore. After 10-min etching, the length of the nanoporous Si nanopillars increased to 15.1 μm, and the thickness of the nanoporous base was reduced even more. The nanoporosity of the nanopillars is more clearly shown in the cracked pillars (Figure 2b,d,f). It is also interesting to note that the nanoporous layer underneath the pillars is thicker than the nanoporous layer directly below the Au film after 6- and 10-min etching (Figure 2d,f).

**Figure 3:**
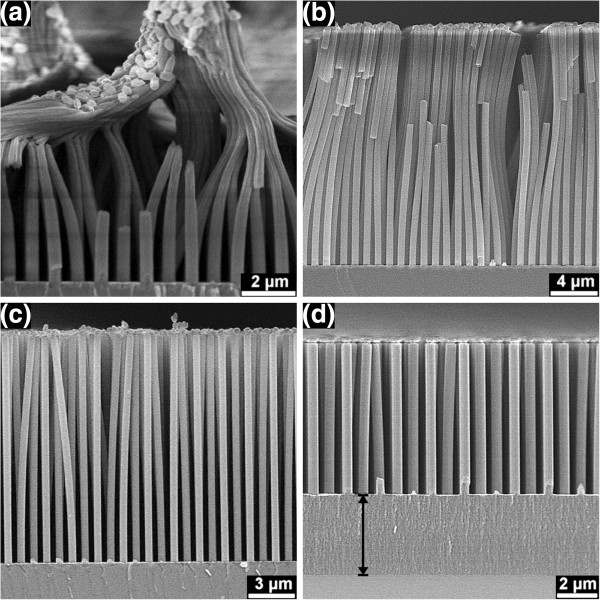
**SEM images of nanopillars formed from the highly doped Si after 10-min etching.** In **(a) ***λ*_1_, **(b) ***λ*_2_, **(c) ***λ*_3_, and **(d) ***λ*_4_ solutions. The distance mark in (**d**) indicates the range of the nanoporous base layer under the Au film and nanopillars.

The highly doped Si was etched for 10 min in solutions with different values of the molar ratio *λ*, and the formed nanopillars are shown in Figure 3. Relatively long nanopillars and a thin nanoporous base layer were observed after etching in the *λ*_1_, *λ*_2_, and *λ*_3_ solutions, while shorter nanopillars and a thick homogenous nanoporous base layer with a thickness of 4.3 μm below the pillars were observed after etching in the *λ*_4_ solution. The nanoporosity of the nanopillars etched in the *λ*_1_, *λ*_2_, and *λ*_4_ solutions becomes obvious in the cracked pillars (Additional file 1: Figure S2). After 10-min etching in the *λ*_1_ and λ_2_ solutions (Additional file 1: Figure S2a,b), it was also observed that the nanoporous base layer below the pillars is thicker than that directly below the Au film. The nanopillars are strongly bent and bonded together at the top after etching in the *λ*_1_ solution (Figure 3a). The bonded nanopillars at the top can be clearly seen in the magnified SEM image (Additional file 1: Figure S3). In addition, the thickness of these nanopillars is about 50% smaller at the top compared to the bottom of the pillars. The bonded and bent nanopillars were also observed after etching in the *λ*_2_ solution (Figure 3b), but they are less bent than those after etching in the *λ*_1_ solution. The nanopillars etched in the *λ*_1_ solution were bonded as bundles, while the nanopillars etched in the *λ*_2_ solution were bonded in rows (Additional file 1: Figure S4a,b). The same thickness is seen both at the top and bottom of the nanopillars etched in the *λ*_2_ solution. Long isolated nanopillars without bending were observed after etching in the λ_3_ solution (Figure 3c). The dependence of the bonding and bending phenomena on the value *λ* is more clearly seen in the tilted SEM images (Additional file 1: Figure S4).

**Figure 4:**
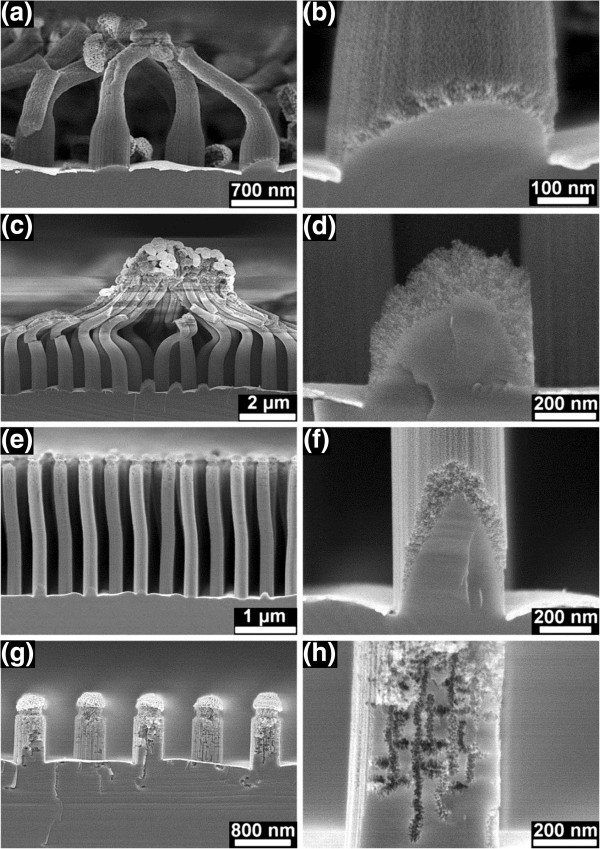
**SEM images of nanopillars formed from the lightly doped Si after 10-min etching.** In **(a, b) ***λ*_1_, **(c, d) ***λ*_2_, **(e, f) ***λ*_3_, and **(g, h) ***λ*_4_ solutions. Panels **b**, **d**, **f**, and **h** show the cracked nanopillars. These cracks were formed during the breaking of the samples for the SEM investigations.

**Figure 5:**
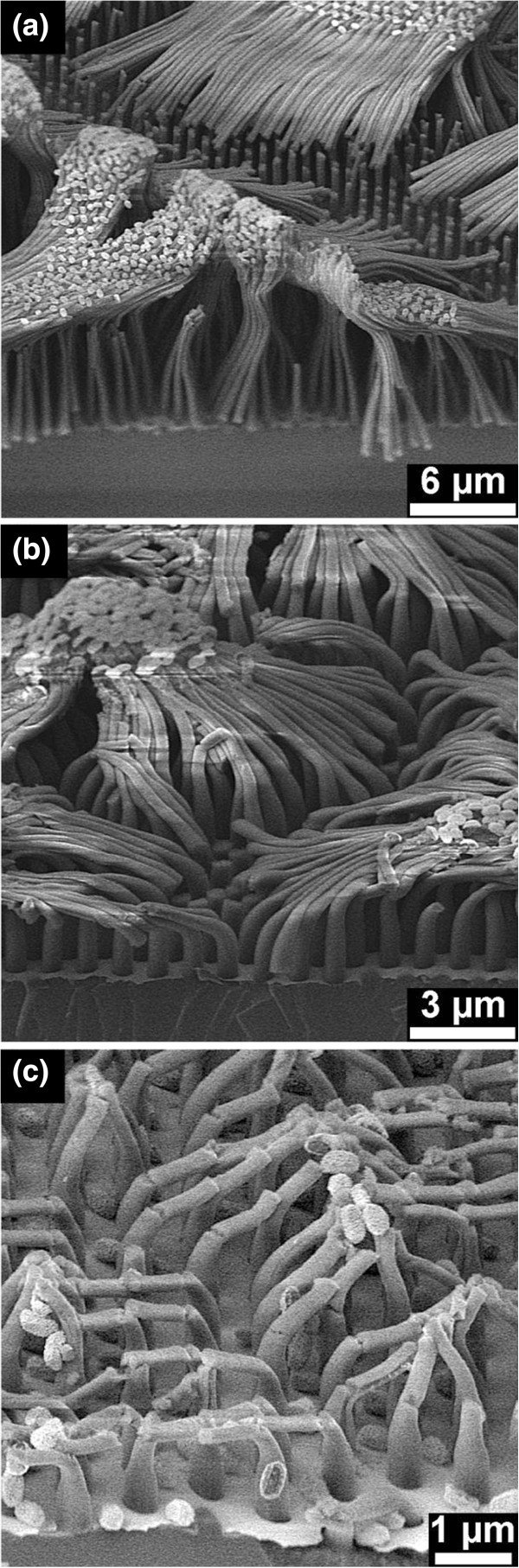
**SEM images of the fractured and cracked Si nanopillars. (a)** Formed from the highly doped Si after etching in *λ*_1_ solution for 10 min, **(b)** from the lightly doped Si after etching in *λ*_2_ solution for 10 min, and **(c)** from the lightly doped Si after etching in *λ*_1_ solution for 10 min.

**Figure 6:**
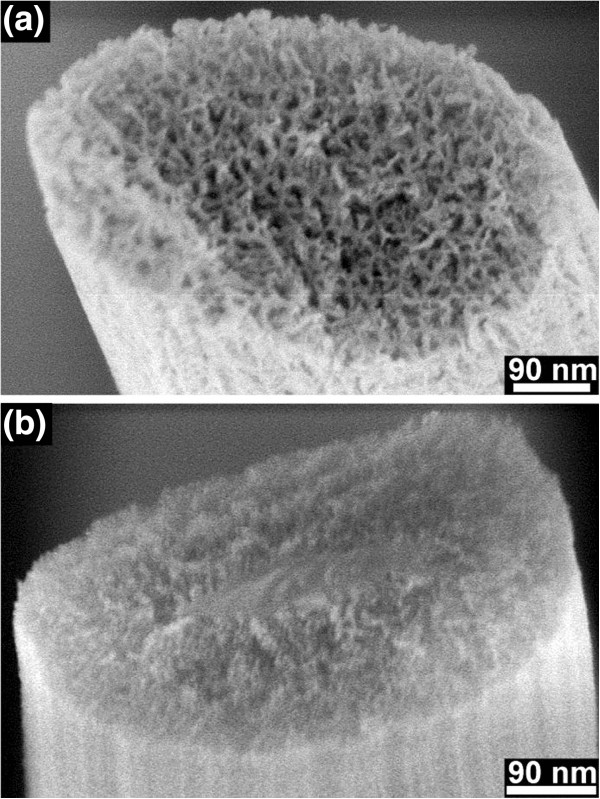
**SEM images of the cross-sectioned nanopillars. (a)** Nanoporous Si nanopillars formed from the highly doped Si, and **(b)** Si nanopillars with solid core and nanoporous shell formed from the lightly doped Si after etching in *λ*_3_ solution for 10 min.

The lightly doped Si was etched for 10 min in solutions with different values of *λ*, and the formed nanopillars are shown in Figure 4. The etching in the *λ*_1_ solution was not homogenous, and at some places, only a nanoporous base was etched underneath the Au film, while at other places, nanopillars with a nanoporous base were observed, and somewhere else, nanopillars without a nanoporous base layer were observed (Additional file 1: Figures S5 and S6). The nanopillars were strongly bonded together at the top and strongly bent after etching in the *λ*_1_ and *λ*_2_ solutions (Figure 4a,c). The thickness on top of the nanopillars is reduced to about 40% and 55% after etching in the *λ*_1_ and *λ*_2_ solutions for 10 min. During etching, the strong bonding and bending caused fracture (as seen between the bundles in Figure 5a,b) and cracking (shown in Figure 5c) of the nanopillars. Further SEM investigations confirmed that these fractures and cracks have been formed during etching, but not due to the sample breaking for the SEM investigation. Slightly double bent, but isolated nanopillars were observed after etching in the *λ*_3_ solution (Figure 4e), while straight and short nanopillars were observed after etching in the *λ*_4_ solution (Figure 4g). The Si nanopillars which formed after etching in the *λ*_1_, *λ*_2_, and *λ*_3_ solutions possess nanoporous shells, and this can be clearly seen in the magnified SEM images (Figure 4b,d,f). It was also observed that the thickness of the shell increased from the bottom to the top of a pillar (Figure 4d,f). Figure 6 shows a cross-sectioned nanoporous Si nanopillar formed from the highly doped Si and a cross-sectioned Si nanopillar with nanoporous shell formed from the lightly doped Si for comparison.

**Figure 7:**
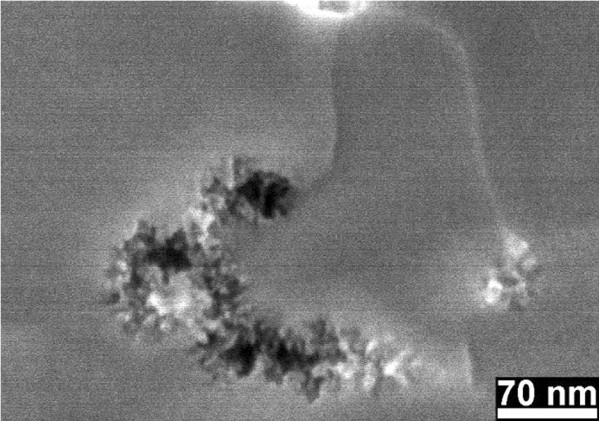
**Magnified SEM image of the bundles of pores under the right nanopillar in Figure**4**g.** Formed from the lightly doped Si after etching in the *λ*_4_ solution for 10 min.

The pore size is clearly influenced by the doping level: around 10 nm of the nanoporous nanopillars formed from the highly doped Si, and around 4 nm of the porous shells of the nanopillars formed from the lightly doped Si. The molar ratio *λ* has almost no influence on the pore size by formation of porous pillars in the highly doped Si. The pore size in the porous shells formed in the lightly doped Si also almost does not change with molar ratio from *λ*_1_ to *λ*_3_. However, some chains of pores with relatively large pore size (around 10 nm) were formed in the lightly doped Si after etching in *λ*_4_ solution for 10 min (Figure 4g,h). Some pores were also observed underneath the Au film (Figure 4g and the corresponding magnified image in Figure 7). This means that the pore formation for the lightly doped Si in the *λ*_4_ solution is not homogenous, and in Figure 7, it is clearly seen that there are channels between the bundles of pores and the surface of the Au film. The pore formation is generally more active in the highly doped Si. Besides, nanoporous layers were even formed at the back side of all Si samples (Additional file 1: Figure S7), and only the front side of the samples was coated with Au film.

**Figure 8:**
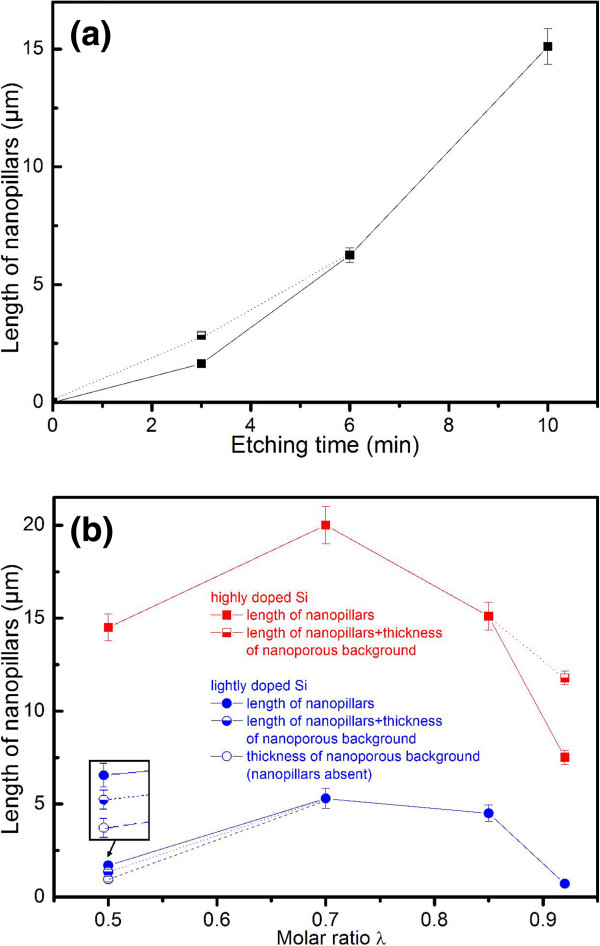
**Nanopillar length as a function of etching time and molar ratio. (a)** The length of the nanopillars as a function of etching time for the highly doped Si in the *λ*_3_ solution and **(b)** the length of the nanopillars as a function of molar ratio *λ* for both highly doped Si (square symbols) and lightly doped Si (circular symbols) with a constant etching time of 10 min. Fully filled symbols indicate the length of the nanopillars, half-filled symbols indicate the total length of the pillars and the thickness of the nanoporous base layer, and unfilled symbols indicate the thickness of the nanoporous base layer in the absence of nanopillars. The inset in (**b**) is a magnified view of the position indicated by the arrow.

Figure 8a shows the length of the nanopillars formed from the highly doped Si in the *λ*_3_ solution as a function of etching time. The length increases with etching time in a nonlinear manner. Figure 8b shows the nanopillar length as a function of the molar ratio *λ*. After 10-min etching, the pillar length varies from 7.5 to 20 μm for the highly doped Si in solutions with different molar ratio *λ*, while the pillar length varies from 0.7 to 5.3 μm for the lightly doped Si (Figure 8b). The etching rate of the highly doped Si is clearly higher than that of the lightly doped Si. The etching rate reaches its maximum at *λ*_3_ for both highly doped Si and lightly doped Si.

## Discussion

It is generally accepted that chemical or electrochemical reactions take place near the noble metal during MaCE [11,13,14]. The Au film can be regarded as cathode and the Si as anode, and the possible reactions are as follows:

(1) $$\mathrm{Cathode}:H_{2}O_{2}+{2H}^{+}\to {2H}_{2}O+{2h}^{+}$$

(2) $$\mathrm{Anode}:\mathrm{Si}+{{4\mathrm{HF}}_{2}}^{-}\to {{\mathrm{SiF}}_{6}}^{2-}+H_{2}↑+2e^{-}$$

A charge transfer is required for the dissolution of Si, and hole (h^+^) injection is an important charge transfer process by MaCE. The electrochemical potential of H_2_O_2_ is much more positive than the valence band of Si, and hole injection from H_2_O_2_ into the valence band is energetically possible [14]. However, the etching rate of H_2_O_2_/HF solution is very low (<10 nm/h) [25], and the noble metal acts as catalyst for the hole injection and thereby improves the etching rate dramatically [11]. Holes are generated at the Au surface by the cathode reaction and injected into the valence band of Si. Normally, the Si electronic bands will equilibrate by contacting the Si surface to the liquid solution and forming an energetic barrier to hinder the charge transfer across the Si/solution interface [26]. Charge transfer is much easier at the metal/solution interface and the metal/semiconductor interface than at the semiconductor/solution interface [25]. Besides, the work function of Au is close to the Femi level of p-type Si, and this also facilitates a charge transfer due to the quasi-ohmic Au/p-Si contact with low barrier.

By the anodic (or electrochemical) etching of Si in a HF-containing solution, electropolishing can be regarded as a reaction limited by the diffusion of HF, and electrochemical pore formation as a reaction limited by the charge supply from the electrode [25]. The transition from the charge-supply-limited reaction to HF-diffusion-limited reaction is characterized by the critical current density *J*_ps_, and electropolishing requires high current densities in excess of *J*_ps_. In this work, the observations of polishing (marked as vertical etching of nanopillars or vertical movement of the Au film front) at the Au film front and pore formation in the formed nanopillars, underneath the Au film and on the metal-off back side of the Si, indicate that charge transfer took place at these sites (interface between the Au film and Si and interface between the Si and solution). In other words, the Au film serves as cathode, and the Si underneath the Au film, the Si pillars, and the back side of the Si wafers can be regarded as anodes. Charge transfer with the highest current density obviously takes place at the Au film front where the holes are generated.

At the Au film front, both polishing and pore formation occurred almost simultaneously for the highly doped Si. Maybe pore formation underneath the pillars is occurring even before polishing (Figure 2d,f and Additional file 1: Figure S2a,b). It is supposed that dopants serve as nucleation sites for pore formation, and the higher doping level leads to a larger thermodynamic driving force for pore formation in the p-type Si [15]. The charge supply (hole injection) is dependent on the concentration of H_2_O_2_ by MaCE, as shown in Equation 1. In the *λ*_1_, *λ*_2_, and *λ*_3_ solutions with relative higher charge supply, only a thin porous base layer is observed (Figure 2f and Additional file 1: Figure S2a,b), and the polishing effect is very strong (indicated by the long pillar length as seen Figure 8b). The thickness of the thin porous base layer is not homogenous, and a thicker layer was generally observed underneath the pillars, where the local current density is smaller than that directly under the Au film. As the molar ratio *λ* increases to 0.92 (*λ*_4_) with small H_2_O_2_ concentration, thick porous base layers (Figure 3d) under the Au film front were observed in the highly doped Si. The current density at the Au film front is reduced by the limited charge supply, and thereby, the polishing is depressed and the formation of pores under the Au film front becomes more active. This is also confirmed by the smaller pillar length compared with pillars etched in the *λ*_1_, *λ*_2_, and *λ*_3_ solutions (as seen in Figure 8b). A thick porous base layer was also observed under the Au film front after 3-min etching in the *λ*_3_ solution (Figure 2a), while the thickness of the porous base layer is reduced with increasing etching time (Figure 2d,f). The polishing effect becomes stronger after the first 3-min etching (Figure 8a). The initial weak polishing effect and the active formation of a thick porous base layer are probably due to the depressed charge transfer by the native SiO_2_ layer and solid by-products of RIE between the Au film and the Si.

At the Au film front, only polishing occurred with the lightly doped Si (Figure 4d,f) in the *λ*_2_ and *λ*_3_ solutions. Pore formation on the sidewalls of the pillars was followed by polishing. It can be imagined that a small amount of holes diffuses from the Au film to the outer surface of the nanopillars, leading to the formation of a nanoporous shell. The nanoporous shell is thicker at the upper side of the pillars (Figure 4d,f) due to the longer time for pore formation at these positions than at the ‘fresh’ bottom. For the *λ*_4_ solution with small H_2_O_2_ concentration, the polishing effect was also suppressed (reduced pillar length in the *λ*_4_ solution as seen in Figure 8b), and pore formation is active (as seen in Figure 4g and 7) due to the low current density. However, the thermodynamic driving force for pore formation is smaller in the lightly doped Si, and only few bundles of pores were observed (Figures 4g and 7). The transition from polishing to pore formation is more obvious in the lightly doped Si, while pore formation is much more active in the highly doped Si. The formation of lightly double-bent nanopillars (Figure 4e) is probably due to the periodic depletion of H_2_O_2_ at the etching front (Au film front), and the corresponding periodic oscillations of the cathodic current can switch the etching directions [19]. It is still unclear why the inhomogeneous etching occurred with lightly doped Si in the *λ*_1_ solution (Additional file 1: Figures S5 and S6). However, this indicates that the current density was not homogenous over the whole Au film during etching in the *λ*_1_ solution.

The etching rate is dependent on the value of *λ* and reaches its maximum at *λ* = 0.7 for both lightly and highly doped Si (Figure 8b). Chartier et al. have systematically studied the dependence of the etching rate on *λ*[12]. As *λ* increases from small values to large values, the reaction changes from HF-concentration-controlled to H_2_O_2_-concentration-controlled, and the value of *λ* between 0.7 and 0.9 is optimized for high etching rate. The etching rate of highly doped Si is clearly higher than that of lightly doped Si, and this phenomenon was also observed in the work of Qu et al. [27]. This is probably due to the higher current density in the highly doped Si. However, the etching rate in this work is clearly higher than that in the work of Qu et al. [27], and this is because a much higher concentration of the total active chemicals in the solution (([HF] + [H_2_O_2_) / [H_2_O] = 1/5) was used here.

Pillar thinning was observed in both highly and lightly doped Si after etching in the *λ*_1_ or *λ*_2_ solution with higher H_2_O_2_ concentration (Figures 3a and 4a,c). It is supposed that oxidation occurred, and then, pillar thinning followed by removing the formed SiO_2_ via HF. The possible oxidation reaction is as follows:

(3) $${\mathrm{Si}}^{0}\;+{2H}_{2}O_{2}\to {\mathrm{SiO}}_{2}\;{+2H}_{2}O$$

Higher oxygen coverage with higher amount of Si-O-Si bridge-bonded oxygen was observed when the H_2_O_2_ concentration is increased by the HF-H_2_O_2_ treatment on the Si surface [28]. Nevertheless, the etching rate of naked Si (without metal coat) is smaller than 10 nm/h in HF/H_2_O_2_ solutions [25]. The thinning or etching rate observed here is clearly higher than that value, indicating that the oxidation is a charge-transfer (or electrochemically)-aided process. The SEM image of the thinned top of the pillars (Additional file 1: Figure S3) suggests that some oxides remain immediately after MaCE. This is also confirmed by the overcharge effect during SEM investigation. However, the pillar thinning or charge-transfer-aided oxidation occurs only in the solutions with high H_2_O_2_ concentrations. Pillar thinning was observed mainly at the top of the pillars because the H_2_O_2_ concentration is higher at the top than at the bottom. For the latter, most of the H_2_O_2_ is consumed for hole injection.

The pillar thinning was found to be always accompanied by pillar bonding and bending. The pillar surface will change from hydrophobic to hydrophilic when Si is oxidized. Therefore, the capillary force becomes more significant when the surface is coated with an oxide layer. Gas bubbles are formed by MaCE (as seen in Equation 2), and the liquid is disturbed locally by the gas bubbling. The surface-oxidized pillars then were bent due to capillary forces. When the top regions of some pillars come into contact, bonding occurs due to the charge-transfer-aided reaction. Both bending and bonding are so strong that fracture or cracking occurs by proceeding MaCE (Figure 5). Besides that, a lower value of *λ* (or higher H_2_O_2_ concentration) for causing the effects of pillar thinning, bending, and bonding is required for highly doped Si. This is probably due to the higher etching rate and the corresponding higher consumption of H_2_O_2_ for highly doped Si.

## Conclusions

In summary, the fabrication of ordered nanoporous Si nanopillar arrays with and without nanoporous base layers and ordered Si nanopillar arrays with nanoporous shells is demonstrated. Pore formation is much more active in the highly doped Si, and the transition from polishing to pore formation is much clearer in the lightly doped Si. Higher etching rates are observed in the Si with higher doping level. Pillar thinning and oxidation are only observed for etching in the solutions with small values of *λ*. Strong bonding and bending of the pillars occur when the surface of the pillars is oxidized. These results help in understanding the MaCE mechanisms. Furthermore, this synthesis has a potential for applications in optoelectronics, sensors, and Li-ion batteries.

## Abbreviations

MaCE: metal-assisted chemical etching; RIE: reactive ion etching; SCIL: substrate conformal imprint lithography; SEM: scanning electron microscope.

## Competing interests

The authors declare that they have no competing interests.

## Authors’ contributions

DW and PS conceived, designed, and analyzed the experiments. RJ performed the substrate conformal imprint lithography. DW, SD, and AA carried out and organized the other experiments. DW and PS wrote the manuscript. All authors discussed the results, commented on the manuscript, and read and approved its final version.

## Authors’ information

DW is a staff scientist at TU Ilmenau. SD is a student at TU Ilmenau. AA is the head of the laboratory (Center for Micro- and Nanotechnologies) at TU Ilmenau. PS is a professor at TU Ilmenau. RJ is an application engineer at SÜSS MicroTec.

## Supplementary Material

Additional file 1 **Supporting information.** Ordered arrays of nanoporous silicon nanopillars and silicon nanopillars with nanoporous shells. Click here for file
