# Supplementary material for: Ordered arrays of nanoporous silicon nanopillars and silicon nanopillars with nanoporous shells
Source: Nanoscale Res Lett. 2013 Jan 21;8(1):42. doi: 10.1186/1556-276X-8-42 (PMC3570473; doi:10.1186/1556-276X-8-42)
Supplement: Additional file 1 — Supporting information. Ordered arrays of nanoporous silicon nanopillars and silicon nanopillars with nanoporous shells. [file 1556-276X-8-42-S1.pdf]

Supporting Information

Ordered Arrays of Nanoporous Silicon Nanopillars and Silicon Nanopillars with Nanoporous Shells

Dong Wang,[1*] Ran Ji,[2] Song Du,[1] Arne Albrecht,[3] and Peter Schaaf[1*]

[1] Chair Materials for Electronics, Institute of Materials Engineering and Institute of Micro- and Nanotechnologies MacroNano®, Ilmenau University of Technology, Gustav-Kirchhoff-Str. 5, 98693 Ilmenau, Germany

[2] SÜSS MicroTec Lithography GmbH, Schleissheimer Str. 90, 85748 Garching, Germany

[3] Center for Micro- and Nanotechnologies, Ilmenau University of Technology, Gustav-Kirchhoff-Str. 7, 98693 Ilmenau, Germany

* Corresponding authors: tel.: +49 3677 69 3170, fax: + 3677 69 3171, and email: dong.wang@tu-ilmenau.de (Dong Wang); tel.: +49 3677 693611, fax: + 3677 69 3171, and email: peter.schaaf@tu-ilmenau.de (Peter Schaaf).

Supporting Figures

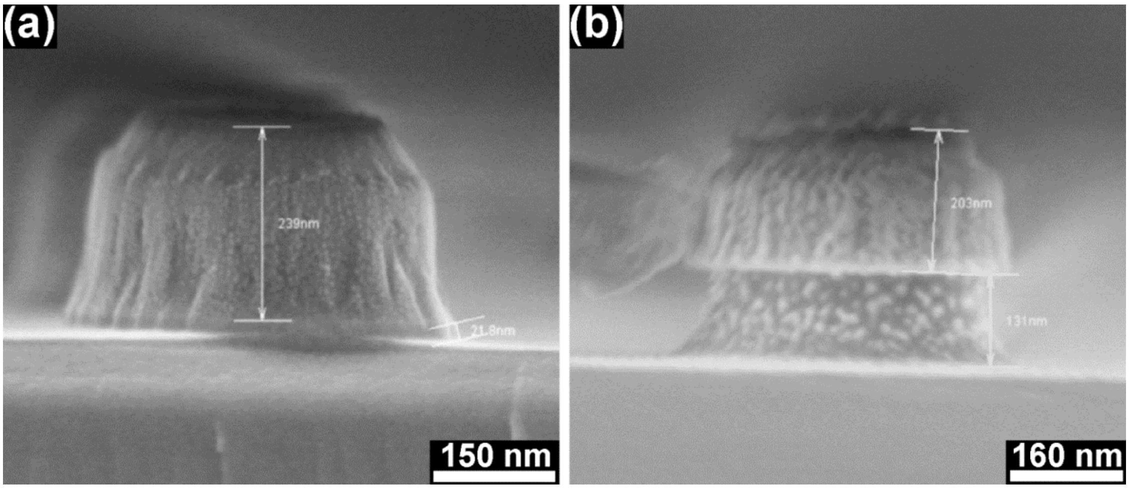

Figure S1. SEM images of the nanopillars after (a) first step ICP-RIE (resist remains 239 nm and 11 nm Si was etched), and (b) 2nd RIE (resist remains 203 nm and 131 nm Si was etched).

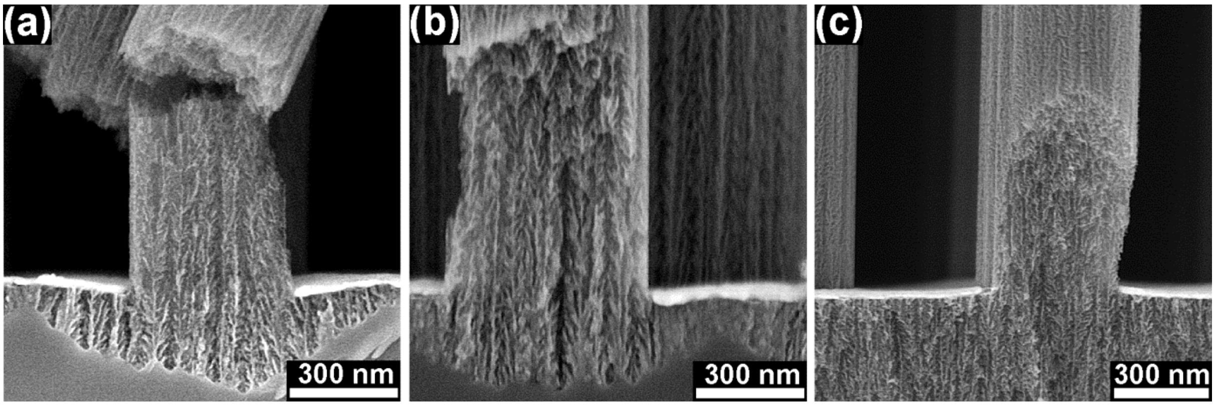

Figure S2. SEM images of the cracked nanoporous Si nanopillars after etching for 10 min in: (a) λ_1-solution, (b) λ_2-solution, and (d) λ_4-solution. These cracks were formed during breaking the samples for the SEM investigation.

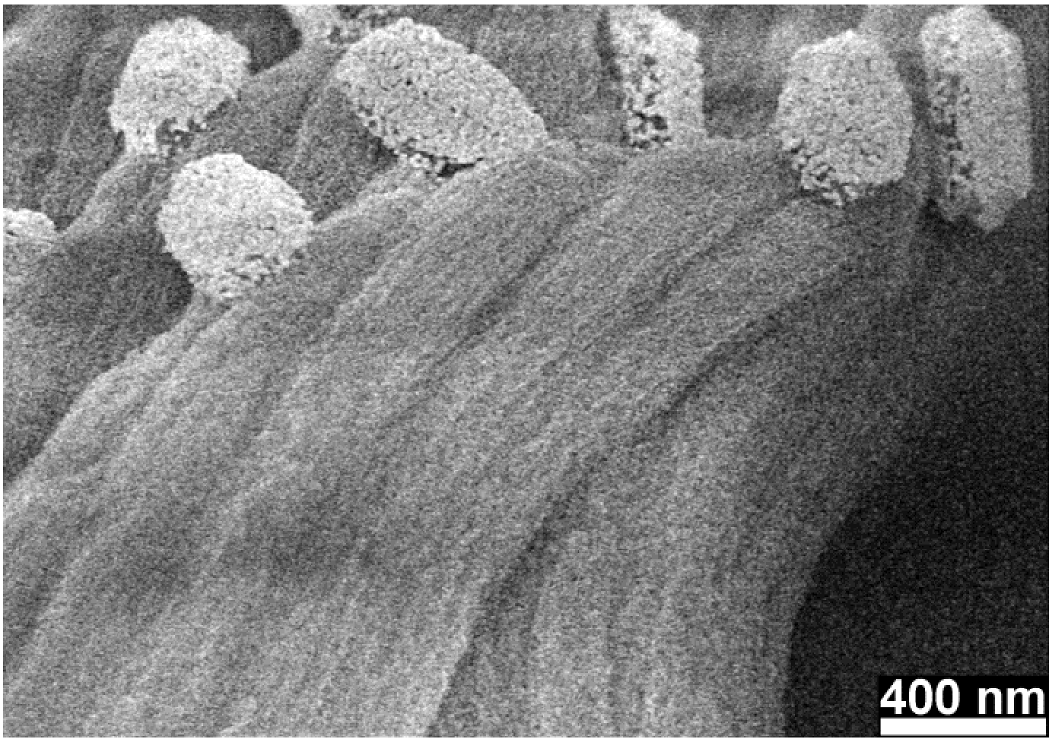

Figure S3. Magnified SEM image of the strongly top-bonded nanopillars formed from the highly doped Si after etching in the λ_1-solution for 10 min. Resist and Au film are not removed.

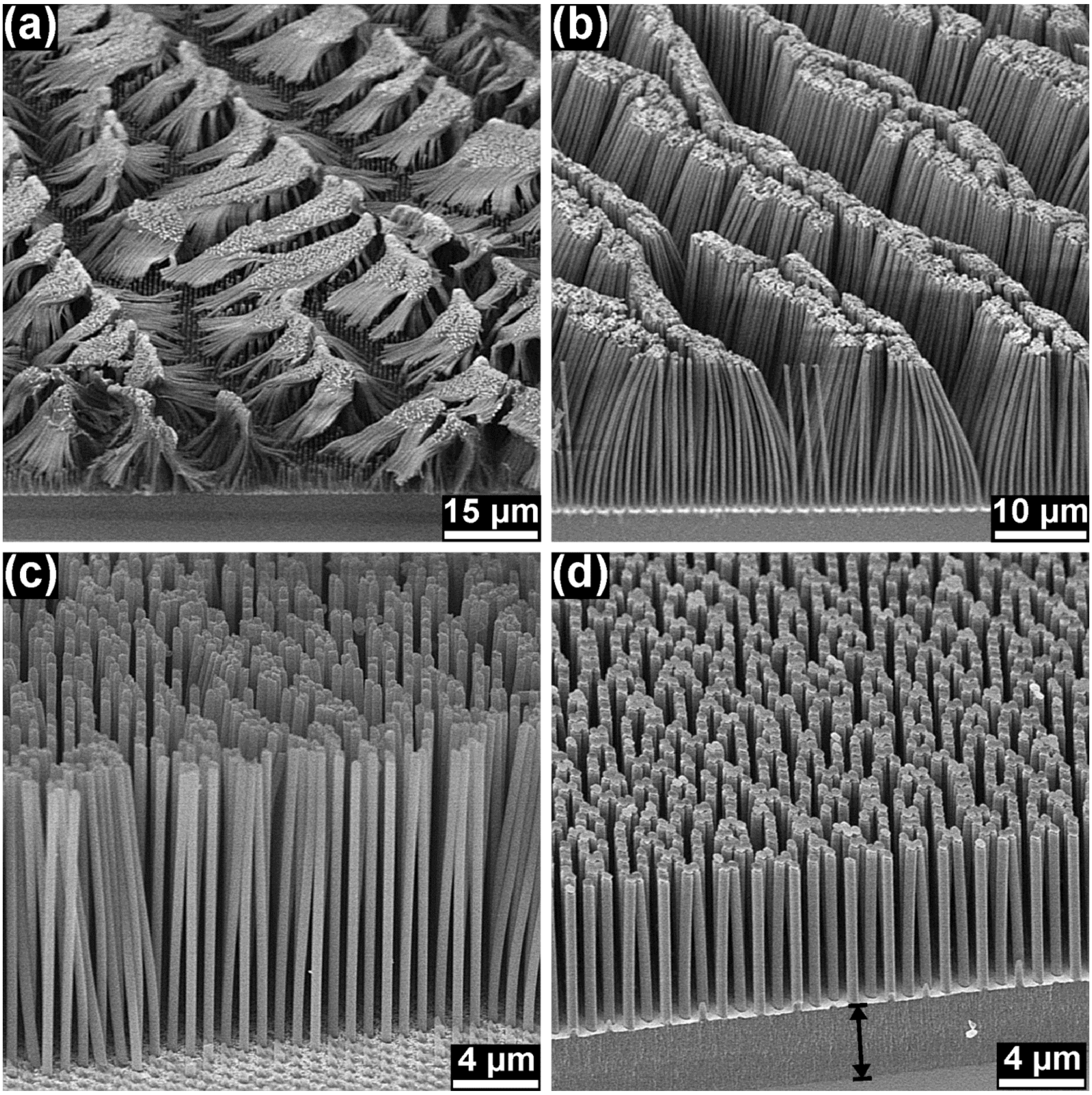

Figure S4. SEM images of the nanoporous Si nanopillars after etching for 10 min in: (a) λ_1-solution, (b) λ_2-solution, (c) λ_3-solution, and (d) λ_4-solution. Distance mark in (d) indicates the range of nanoporous base under Au film and nanopillars.

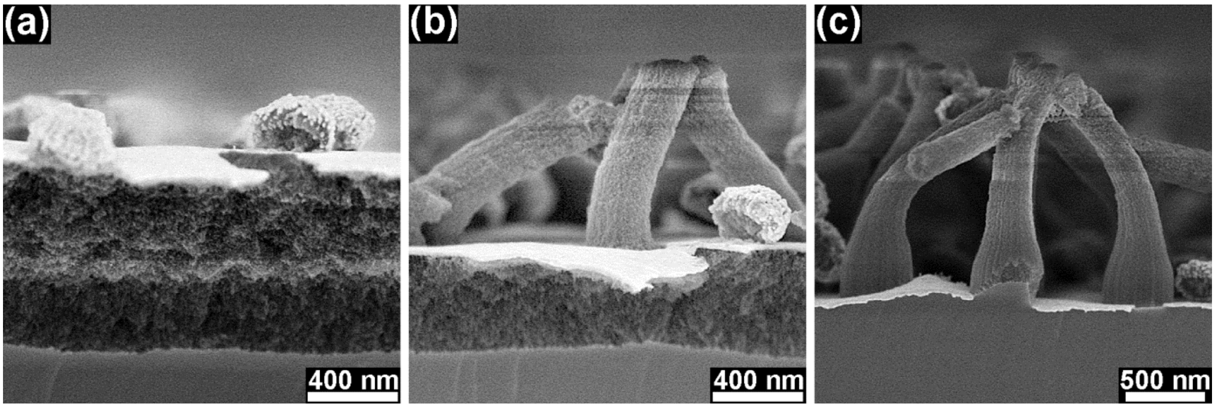

Figure S5. SEM images of the lightly doped Si after etching in the λ_1-solution for 10 min. The etching is inhomogenous and different structural forms were observed: (a) only nanoporous base layer under the Au film (nanopillars absent), (b) nanopillars with nanoporous base layer, and (c) nanopillars without nanoporous base layer.

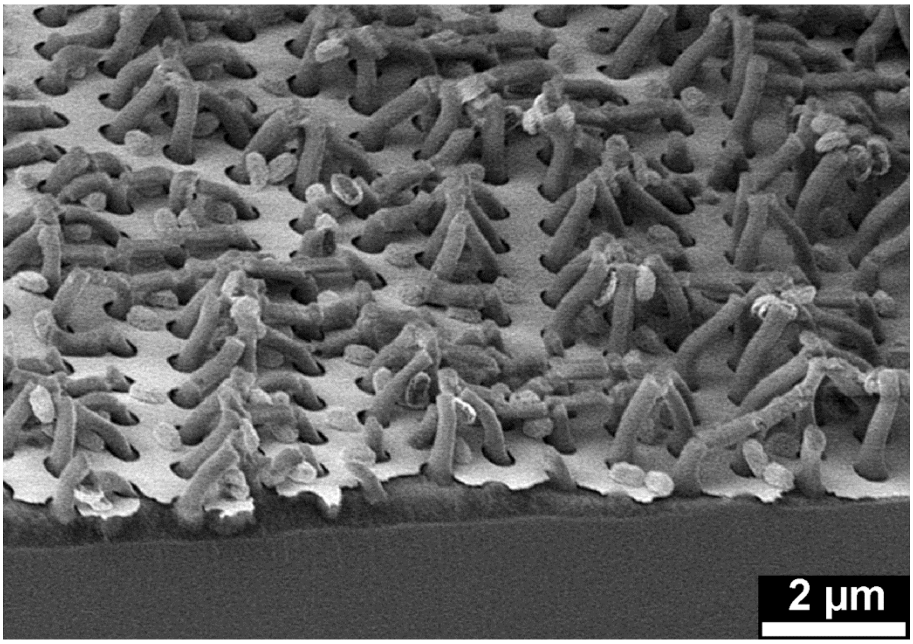

Figure S6. SEM images of the lightly doped Si after etching in the λ_1-solution for 10 min. The thickness of the nanoporous base layer is reduced from left to right, indicating the inhomogenous etching in the λ_1-solution.

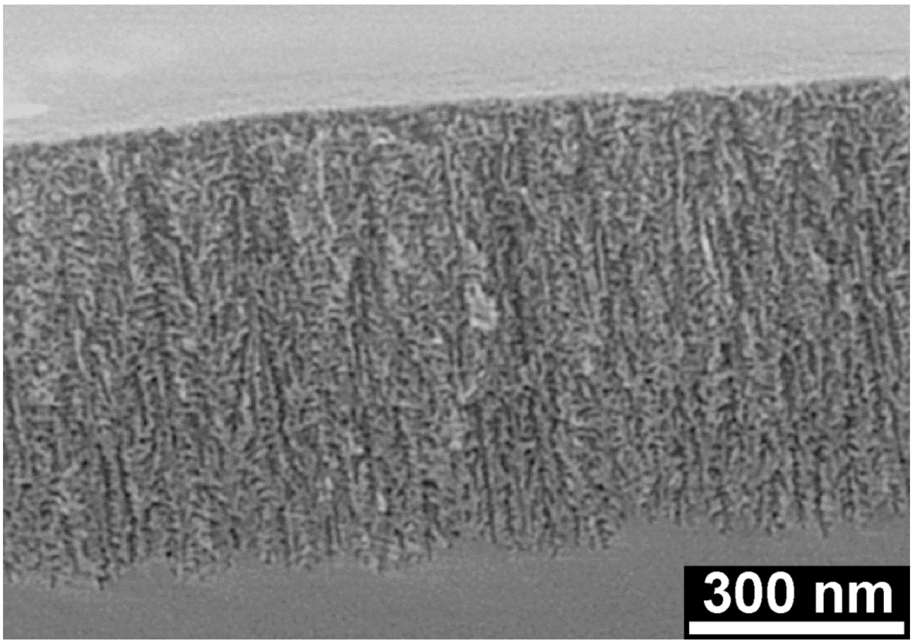

Figure S7. SEM image of the nanoporous layer on the back side of the highly doped Si after etching in the λ_3-solution for 10 min. During metal-assisted chemical etching, only front side of Si was coated with Au film.
